# The Blocking on the Cathepsin B and Fibronectin Accumulation in Kidney Glomeruli of Diabetic Rats

**DOI:** 10.1155/2015/812825

**Published:** 2015-05-18

**Authors:** Aleksandra Wyczalkowska-Tomasik, Irena Bartlomiejczyk, Agnieszka Wirkowska, Lukasz Koperski, Barbara Gornicka, Leszek Paczek

**Affiliations:** ^1^Department of Immunology, Transplant Medicine and Internal Diseases, The Medical University of Warsaw, 02-006 Warsaw, Poland; ^2^Department of Pathology, The Medical University of Warsaw, 02-004 Warsaw, Poland

## Abstract

Hyperglycemia results in the activation of tissue angiotensin II. Angiotensin II stimulates the synthesis of ECM proteins and causes a decrease activity of proteolytic enzymes. The aim of this study was to assess the impact of multilevel blocking of the RAAS, cathepsin B activity, and fibronectin accumulation in the glomerular in the rats diabetes model. Sixty male Wistar rats were initially included. Diabetes was induced by intravenous administration of streptozotocin. The animals were randomized to six groups of ten rats in group. Rats in the four groups were treated with inhibitors of the RAAS: enalapril (EN), losartan (LOS), enalapril plus losartan (EN *+* LOS), and spironolactone (SPIR); another group received dihydralazine (DIH) and the diabetic rats (DM) did not receive any drug. After six weeks, we evaluated blood pressure, 24 h urine collection, and blood for biochemical parameters and kidneys. In this study, fluorometric, ELISA, and immunohistochemical methods were used. Administration of EN *+* LOS increased activity of cathepsin B in homogenates of glomeruli compared to DM. Losartan treatment resulted in reduction of the ratio kidney weight/body weight compared to untreated diabetic rats. SPIR resulted in the increase activity of cathepsin B in the homogenate of glomeruli. The values of cathepsin B in the plasma of rats in all studied groups were similar and showed no tendency.

## 1. Introduction

Diabetes mellitus is a serious social problem. According to the World Health Organization (WHO), in 2030 the number of people with diabetes worldwide will increase to 360 million, representing 4.5% of the global population [1].

Diabetic nephropathy is the most frequent complication of diabetes that develops in up to 30–40% of patients. The main change of diabetic nephropathy is a thickening of the glomerular basement membrane and expansion of ECM proteins.

Hyperglycemia results in the activation of tissue angiotensin II, which plays an important role in the pathogenesis of kidney disease, through inflammation, fibrosis, vascular wall remodeling, and oxidative stress [2]. It was shown that blocking the AT1 receptor and angiotensin II-converting enzyme reduces the levels of inflammatory factors (NF-kB, IL-6, and TNF-*β*1) and is responsible for the processes of fibrosis (CTGF and TGF-*β*1) [3, 4].

Fibronectin is a glycoprotein present in the extracellular matrix, basal membranes, plasma, and other body fluids. FN and its receptors regulate many cellular functions. The gene expression of FN in tissues of healthy adults is generally low and increases in areas of wound healing and damaged tissue. Hyperglycemia causes increased expression of genes responsible for synthesis of FN [5, 6].

Cathepsin B (EC 3.4. 22.1) belongs to a class of cysteine proteinases and has optimum activity in acidic environment but can also be active in other pH ranges [7]. It participates in numerous physiological and pathological processes. It degrades structural proteins and enzymes in the cell, degrades the main elements of the basement membrane, and activates proenzymes, hormones, and growth factors involved in the induction phase and the executive apoptosis [8]. Glomerular homogenates of healthy rats show high proteolytic activity of cathepsins. It has been shown that the decrease of the activity of proteolytic enzymes in the states of hyperglycemia is genetically determined as type 2 diabetes in rats and in isolated glomeruli of diabetic rats [9, 10].

The aim of this study was to assess the impact of multilevel blocking of the RAAS using an ACE inhibitor, AT1 receptor antagonist, administered separately and together, an inhibitor of aldosterone on the activity of cathepsin B, and fibronectin accumulation in the glomerular in the course of diabetic nephropathy in the diabetes model in rats.

## 2. Material and Methods

This study was performed in accordance with the Ethical Committee Affairs Experiments on Animals of the Medical University of Warsaw (Opinion number 5/2006). Sixty male Wistar rats weighing 180–200 g were initially included. Diabetes was induced by intravenous administration of streptozotocin [8]. After one week, blood glucose levels were evaluated. The animals were randomized to 6 groups of 10 rats in group. Rats in the 4 groups were treated with inhibitors of the RAA system, drugs were administered in drinking water in the morning: EN: enalapril 3.2 mg/kg/day; LOS: losartan 15 mg/kg/day; EN + LOS: enalapril and losartan, respectively, 3.2 and 7.5 mg/kg/day; SPIR: spironolactone 15 mg/kg/day. Another group received DIH, dihydralazine, 2.7 mg/kg/day and the DM group did not receive any drug.

The animals were followed up for six weeks (five weeks, the duration of action of drugs), and blood glucose was regularly analyzed. When blood glucose was above 700 mg/dL, insulin was administered at a dose of 0.25–1.0 IU/day. In the last week of the experiment, rats' blood pressure was measured using a pressure sensor APM MK-9301 (MK-Design, USA). A 24 hr urine collection was obtained at the end of the 6-week study using metabolic cages and urinary creatinine and microalbumin were measured. Then urine was frozen at −80°C and stored for future analysis.

The animals were euthanized and kidney and blood were collected. One of the kidneys was stored in saline in an ice bath until the isolation of glomeruli [11]. The second kidney was used for histological examination and placed in buffered formalin. The plasma biochemical tests performed are the following: glucose, total protein, albumin, creatinine, urea, and bicarbonate. Some of plasma destined for further research were frozen at −80°C.

Glomeruli were isolated according to the method developed and described by Spiro [12].

Isolated glomeruli were homogenized with a homogenizer Labsonic U (B Braun, USA).

DNA in homogenates of glomeruli was determined using the fluorometric reagent Bisbenzimide H 33258 (Hoechst, Germany) as previously described [11]. Protein in homogenates of glomeruli was determined spectrophotometrically using the BCA assay (bicinchoninic acid), protein assay reagent (Pierce, Beijerland, Netherlands) [13].

Cathepsin B activity in glomerular homogenates, urine, and plasma was measured fluorometrically using the synthetic substrate Z-Arg-Arg-AMC (N-CO_2_-L-arginyl-arginine-7-amino-4-methylocoumarin salt) (Bachem, Biochemica GmbH, Heidelberg, Germany) as previously described [14].

FN concentration was determined by ELISA (enzyme-linked immunosorbent assay; immunoenzyme test) as previously described [15, 16].

Immunohistochemical staining was performed in paraffin sections of kidney with an antibody against fibronectin (Chemicon International, USA). Immunohistochemical analysis began with the assessment of the entire tissue section to determine representative areas. We evaluated 20 subsequent glomeruli within the renal cortex of representative areas. In relation to the glomerular vascular loops, each immunohistochemical reaction assessment was based on an analysis of two features: the intensity of the antibody reaction and the percentage of immunopositive vascular loops. Immunohistochemical expression was evaluated semiquantitatively using a 4-point scale:

 (0) no reaction or very weak and focal (<1%) expression;
 (1+) low intensity reaction, with moderate intensity expression of <50% of vascular loops and high intensity expression being segmental and in <25% of vascular loops;
 (2+) moderate intensity expression involving >50% of vascular loops and high-intensity expression being segmental or continuous involving 25–75% of vascular loops;
 (3+) high intensity expression that is continuous and includes >75% of vascular loops.

Immunohistochemical expression of all markers tested within the mesangium was evaluated in a similar fashion. The final evaluation for each specimen was the average (rounded to unity) from the analysis of 20 glomeruli.

The results obtained were analyzed using STATISTICA, version 9.0 which is available at the Medical University of Warsaw. Statistical nonparametric tests were used: ANOVA rank Kruskal-Wallis. Obtaining a result of analysis of the level of significance *P* < 0.05 was an indication for the use of post hoc test, Duncan. Statistical inference was performed at a significance level of *P* ≤ 0.05.

## 3. Results

Biochemical characterization of the study groups is presented in Tables 1 and 2.

**Figure 1:**
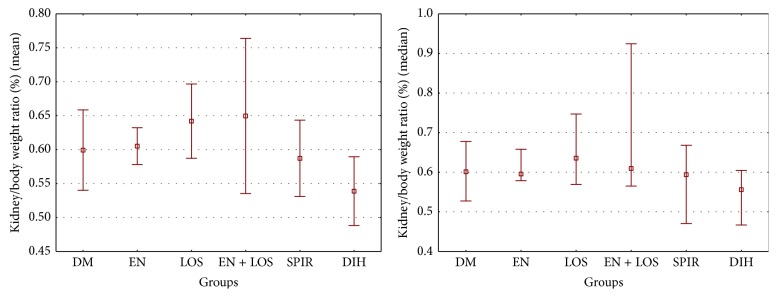
Kidney/body weight ratio in diabetic rats untreated and treated with enalapril, losartan, enalapril and losartan together, spironolactone, or dihydralazine. Results presented as mean ± SD and median (range). Statistically significant differences test of Kruskal-Wallis *P* = 0.0099 has been shown. *P* values calculated in the test and post hoc Duncan: DM versus DIH *P* = 0.01; DIH versus EN *P* = 0.01; DIH versus LOS *P* = 0.0001; DIH versus EN + LOS *P* = 0.01; DIH versus SPIR *P* = 0.04; SPIR versus LOS *P* = 0.03.

The value of kidney weight given as a percentage of final body weight in the group of untreated diabetic rats was 0.60 ± 0.06% and was significantly higher compared to the group of diabetic rats treated dihydralazine (0.54 ± 0.05%, *P* = 0.01). In addition, statistically significant differences were demonstrated between the group of diabetic rats treated dihydralazine and groups with diabetes treated enalapril (*P* = 0.01), losartan (*P* = 0.0001), enalapril and losartan in combination (*P* = 0.01), and spironolactone (*P* = 0.04), and between group of diabetic rats treated with spironolactone and losartan (*P* = 0.03) (Table 3, Figure 1).

**Figure 2:**
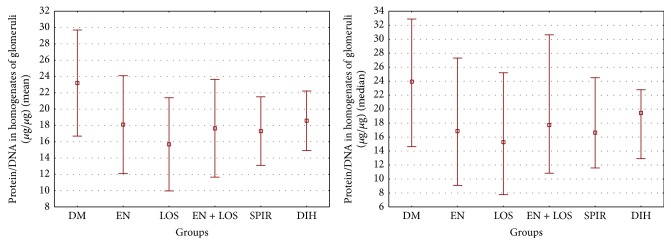
Comparison of the protein/DNA in homogenates of glomeruli in diabetic rats. Results presented as mean ± SD and median (range). *P* values calculated by post hoc test of Duncan were DM versus LOS *P* = 0.01 and DM versus SPIR. *P* = 0.045.

The coefficient protein/DNA in homogenates of glomeruli in the group of untreated diabetic rats was 23.3 ± 6.50 *µ*g/*µ*g and was significantly higher compared to a group of diabetic rats treated losartan 15.68 ± 5.71 *µ*g/*µ*g, *P* = 0.01, and a group of rats treated spironolactone 17.31 ± 4.2 *µ*g/*µ*g, *P* = 0.045 (Table 3, Figure 2).

**Figure 3:**
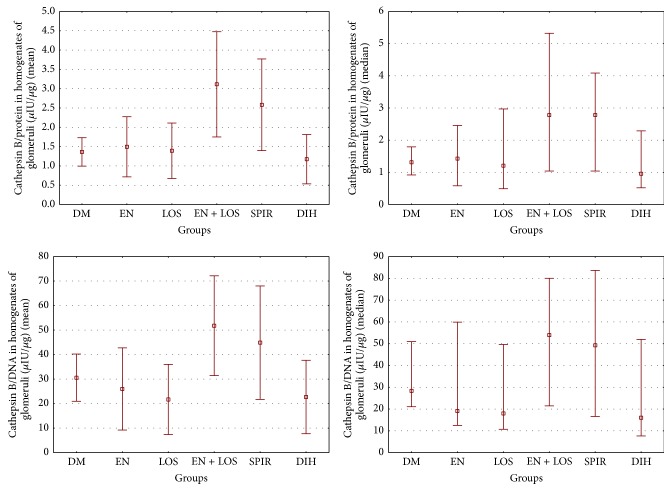
Comparison of cathepsin B activity in homogenates of glomeruli in study groups. Results were presented as mean ± SD and median (range). Activity of cathepsin B expressed per *µ*g protein demonstrated statistically significant differences; ANOVA rang Kruskal-Wallis test *P* value 0.001 and Duncan's post hoc test DM versus EN + LOS *P* = 0.001; DM versus SPIR *P* = 0.02; EN versus EN + LOS *P* = 0.002; EN versus SPIR *P* = 0.03; LOS versus EN + LOS *P* = 0.001; LOS versus SPIR *P* = 0.02; EN + LOS versus DIH *P* = 0.0004; DIH versus SPIR *P* = 0.009. The activity of cathepsin B expressed per *µ*g DNA. ANOVA rang Kruskal-Wallis test *P* value 0.0015 and post hoc Duncan test DM versus EN + LOS *P* = 0.04; EN versus EN + LOS *P* = 0.01; EN versus SPIR *P* = 0.05; LOS versus EN + LOS *P* = 0.01; LOS versus SPIR *P* = 0.02; EN + LOS versus DIH *P* = 0.01; SPIR versus DIH *P* = 0.03.

Activity of cathepsin B in terms of microgram of DNA in homogenates of glomeruli in the group of untreated diabetic rats was 30.60 ± 9.65 *µ*IU/*µ*g and was lower compared to the group of diabetic rats treated with enalapril and losartan in combination (*P* = 0.04) and a group of rats diabetes treated with spironolactone (NS) and, respectively, was 51.79 ± 20.37 and 44.88 ± 32.18 *µ*IU/*µ*g. In the groups, diabetic rats treated with enalapril, losartan, and dihydralazine cathepsin B activity per *µ*g of DNA were insignificantly lower compared with untreated diabetic rats and were, respectively, 25.99 ± 16.75, 21.71 ± 14.32, and 22.70 ± 14.97 *µ*IU/*µ*g. In addition, statistically significant differences were demonstrated between the group of diabetic rats treated with enalapril and losartan together, a group of diabetic rats treated with enalapril (*P* = 0.01), losartan (*P* = 0.01), and dihydralazine (*P* = 0.01), and groups of diabetic rats treated with spironolactone and enalapril (*P* = 0.05), losartan (*P* = 0.02), and dihydralazine (*P* = 0.03). Similar results were obtained converting activity of cathepsin B in homogenates of glomeruli in microgram of protein, Table 3, Figure 3.

**Figure 4:**
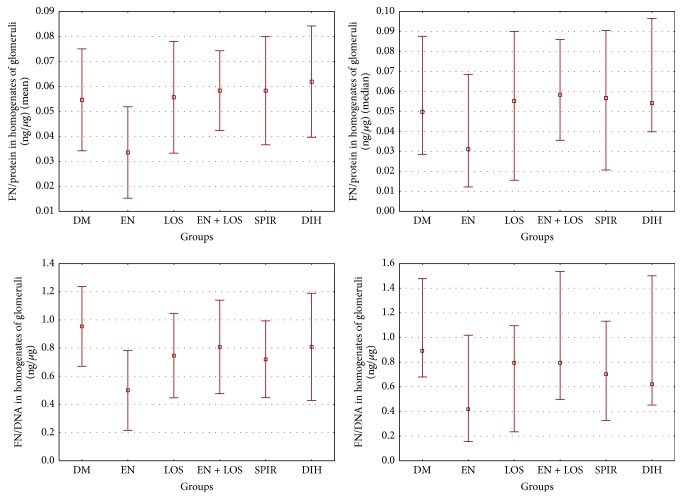
The comparison fibronectin concentration evaluated in glomerular homogenates. Results are presented as mean ± SD and median (range). Data are expressed per *µ*g protein and per *µ*g DNA. Differences between groups were not statistically significant.

Fibronectin concentration values per 1 *μ*g of protein in homogenates of glomeruli in the untreated diabetic rats and the other treated diabetic rats showed no statistically significant differences. Similar results were obtained converting the concentration of fibronectin in glomerular homogenates for the presence of DNA (Table 3, Figure 4).

**Figure 5:**
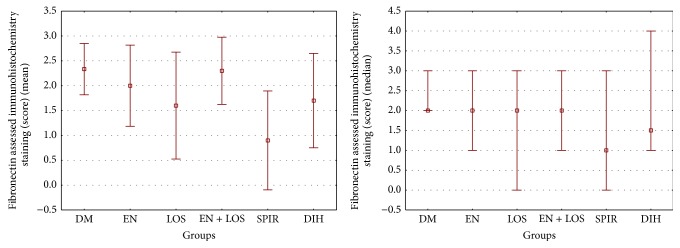
The content of fibronectin (FN) in glomeruli diabetic rats untreated (DM) and diabetic rats treated with enalapril (EN), losartan (LOS), enalapril and losartan together (EN + LOS), spironolactone (SPIR), or dihydralazine (DIH). Results were presented as mean ± SD and median (range). Differences between groups were statistically significant. ANOVA rang Kruskal-Wallis test *P* value = 0.02 and post hoc Duncan: DM versus SPIR *P* = 0.003, SPIR versus EN *P* = 0.02, and SPIR versus EN + LOS *P* = 0.003.

**Figure 6:**
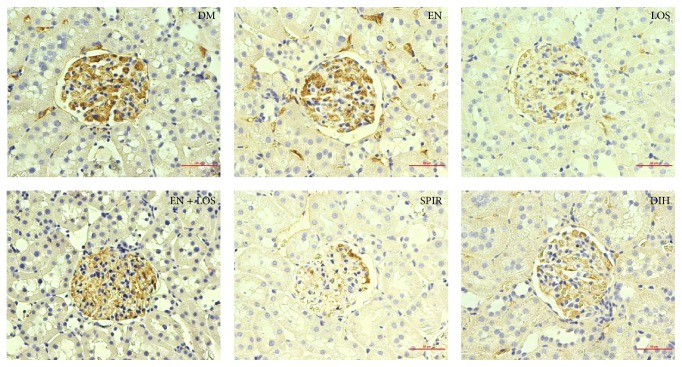
The contents of FN in glomeruli of STZ diabetic rats untreated (DM) and STZ diabetic treated enalapril (EN), losartan (LOS), including enalapril, and losartan (EN + LOS), spironolactone (SPIR), and dihydralazine (DIH), determined by immunohistochemistry staining, 40x magnification, scale 50 *µ*m.

The content of fibronectin in kidney glomeruli evaluated immunohistochemical staining in the untreated diabetic rats was 2.33 ± 0.52 score and was higher compared to the other groups examined, including significantly higher (*P* = 0.003) compared to the diabetic rats treated spironolactone 0.90 ± 0.99 score. Additionally, statistically significant differences have been shown between groups of diabetic rats treated with spironolactone and enalapril (*P* = 0.02) and diabetic rats treated with spironolactone and losartan and enalapril in combination (*P* = 0.003) (Table 3, Figures 5 and 6).

## 4. Discussion

Our study, performed on an animal model of diabetes mellitus induced by streptozotocin, aimed at assessing the impact of RAAS blocking on the activity of cathepsin B and the accumulation of FN. Diabetic rats were randomized into six treatment groups. Rats of groups 1–5 were treated with an ACE inhibitor, AT1 receptor antagonist, administered separately and together, and an inhibitor of aldosterone and dihydralazine. The rats in group number 6 were not treated and they constituted the control group. In the renal cortex of diabetic rats treated and untreated, performed immunohistochemical evaluation of fibronectin was observed. The activity of cathepsin B and the concentration of FN were assessed in homogenates of glomeruli, in 24 hour urine and plasma.

Fibronectin is a protein of the extracellular matrix, produced by the cells and endothelial mesangium [17, 18]. In mild and moderate diabetic nephropathy, accumulation of FN in the matrix mesangium has been shown and increased excretion in the urine does appear up earlier than microalbuminuria [17, 19].

In previous studies, the correlation between the accumulation FN and cathepsin B activity in the kidney glomerulus in diabetic rats has been shown [20].

The genetically determined type II diabetes in rats showed an increased accumulation of fibronectin (FN) in the kidney glomerulus. This phenomenon is accompanied by a decreased ability to degrade this protein [9]. Cumulative increase in ECM proteins in glomeruli depends on the increase of synthesis and/or reduces protein degradation. Proteolytic enzymes are responsible for maintaining the balance of synthesis and degradation of components of the extracellular matrix (ECM) [21].

Onozato et al. [22] showed a decrease of FN immunoreactivity in the kidney glomerulus in rats with hypertension that have blocked aldosterone and angiotensin II. In the present study the contents of the FN, determined by immunohistochemical staining in renal glomeruli of diabetic rats treated with enalapril, losartan, spironolactone, and dihydralazine were lower compared to the amount of FN in the renal glomeruli of rats with untreated diabetes. The largest decrease in the content FN was obtained in the group of diabetic rats receiving spironolactone (*P* = 0.003). No FN obtained reduction in kidney glomerulus of diabetic rats was treated with enalapril and losartan together.

The concentration of fibronectin in glomerular homogenates, based on DNA, in groups of diabetic rats treated has only a slight decrease compared to untreated diabetic rats. Similarly to the fibronectin excretion in the urine per mg creatinine, it has slightly decreased in the groups treated diabetic rats compared to untreated diabetic rats, while the concentration of FN in plasma of rats in all groups examined were in similar extent. Rao et al. [23] giving the rats streptozotocin diabetes ACEi and AT1 blocker received separately and together decrease the concentration of FN in the plasma.

The reduction ratio kidney weight/body weight, reflecting the development of kidney hypertrophy, was obtained only in the group of diabetic rats treated with dihydralazine. The coefficient of protein/DNA, reflecting glomerulosclerosis, was decreased in the group with diabetic rats treated with losartan (*P* = 0.01) and spironolactone (*P* = 0.045). In the other groups treated diabetic rats have shown a downward trend compared to the group of untreated diabetic rats.

A significant reduction of albuminuria has been shown in diabetic rats treated with dihydralazine compared to untreated diabetic rats. In other groups of diabetic rats receiving treatment, a decreasing trend of albuminuria has been shown in relation to untreated diabetic rats. Rao et al. [23] demonstrated reduction in daily urinary albumin excretion in treated diabetic rats compared to untreated diabetic rats.

Angiotensin II stimulates the synthesis of ECM proteins and causes a decrease activity of proteolytic enzymes. Cathepsin B (EC 3.4. 22.1) belongs to a class of cysteine proteinases and plays many functions in physiological and pathological processes. Glomerular homogenates of healthy rats show high proteolytic activity of cathepsins. Reduction of the activity of proteolytic enzymes in the states of hyperglycemia in the genetically determined type 2 diabetes in rats and in streptozotocin diabetic rats has been shown [9, 10, 24].

Multilevel blocking of the RAA system significantly increased activity of cathepsin B in homogenates of glomerular in diabetic rats treated with spironolactone (*P* = 0.02) and in combination with therapy with losartan and enalapril (*P* = 0.001) compared to the untreated diabetic rats. Dihydralazine administration, enalapril, or losartan in rats with diabetes did not lead to the expected results. Suzuki et al. [25] showed a beneficial effect of treatment ACEi to restore the activity of cathepsin B in the heart and Kim et al. [26] demonstrated that combination therapy ACEi and ARB (angiotensin-II receptor blocker) can effectively prevent or reverse myocardial fibrosis. Maione et al. [27] showed that the development of an end-stage renal failure and change micro- to macroalbuminuria were significantly reduced after treatment of ACEi versus placebo and ARB versus placebo. This effect is not present in combined therapy (ACEi + ARB) versus monotherapy.

Assessing the activity of cathepsin B in 24-hour urine has shown a downward trend in the values of individual treatment groups compared to diabetic rats which are not treated. The values of cathepsin B in the plasma of rats in all groups studied were similar and showed no tendency.

Lysosomal enzymes, namely, cathepsins, are responsible for intracellular protein turnover [28]. Primarily its role is to maintain an intracellular pool of amino acids [29].

A part from controlling protein metabolism cathepsin is involved in the process of autophagy.

The summary of autophagy is provided by Deretic [30]. In summary autophagosomes remove irreversibly damaged mitochondria and toxic molecular macromolecules. Recently it is proved that the process of autophagy and activity of cathepsins are strictly linked to the process of inflammation.

## Figures and Tables

**Table 1:** The characteristics of treatment groups.

Groups	Glucose	Creatinine	Urea	HCO_3_	Fibronectin	Cathepsin B
	mg/dL	mg/dL	mg/dL	mmol/L	*μ*g/mL	*μ*IU/mL
DM						
Mean ± SD	746.6 ± 47.5	0.66 ± 0.11	62.6 ± 16.1	25.5 ± 1.1	20.91 ± 4.47	44.61 ± 11.88
Median (range)	763.0 (667.0–793.0)	0.7 (0.5–0.8)	64.0 (43.0–81.0)	25.8 (24.3–26.8)	20.23 (15.06–27.54)	41.03 (21.33–63.85)
EN						
Mean ± SD	706.8 ± 68.8	0.54 ± 0.17	61.6 ± 8.4	27.2 ± 1.8	25.08 ± 1.74	49.6 ± 6.60
Median (range)	703.5 (607.0–812.0)	0.5 (0.3–0.9)	61.5 (48.0–75.0)	27.1 (24.7–29.5)	25.59 (22.62–26.78)	46.57 (43.65–60.46)
LOS						
Mean ± SD	724.7 ± 99.0	0.66 ± 0.13	67.2 ± 15.8	24.7 ± 2.9	21.04 ± 2.52	49.08 ± 10.75
Median (range)	709.0 (577.0–856.0)	0.6 (0.5–0.9)	64.0 (51.0–105.0)	23.1 (21.7–28.3)	20.08 (18.70–24.94)	45.49 (39.07–66.40)
EN + LOS						
Mean ± SD	764.9 ± 61.0	0.57 ± 0.09	75.3 ± 17.7	25.8 ± 2.8	23.58 ± 3.74	50.54 ± 22.79
Median (range)	776.0 (657.0–833.0)	0.5 (0.5–0.7)	71.0 (53.0–116.0)	24.5 (21.9–29.8)	25.08 (16.62–27.06)	48.25 (25.63–80.59)
SPIR						
Mean ± SD	679.0 ± 82.0	0.61 ± 0.12	59.6 ± 10.9	26.1 ± 2.6	22.31 ± 3.02	44.7 ± 7.91
Median (range)	670.0 (570.0–834.0)	0.6 (0.4–0.8)	59.0 (47.0–80.0)	26.5 (22.2–30.0)	23.37 (17.28–25.55)	48.70 (26.88–51.10)
DIH						
Mean ± SD	680.6 ± 159.7	0.64 ± 0.09	59.8 ± 13.8	25.9 ± 2.3	23.87 ± 2.49	38.98 ± 12.90
Median (range)	699.0 (380.0–858.0)	0.6 (0.5–0.8)	63.5 (36.0–75.0)	26.2 (21.6–28.9)	23.95 (20.39–27.74)	37.07 (23.09–56.56)

ANOVA test	NS	NS	NS	NS	NS	NS

The results are presented as mean ± SD and median (range). Statistically significant differences between the groups have been shown, the levels of significance *P* ≤ 0.05 in the 95% confidence interval.

**Table 2:** The values of blood pressure and biochemical parameters evaluated inurine collection.

Groups	Blood pressure	Creatinine clearance	Albuminuria/creatinine	Fibronectin/creatinine	Cathepsin B/creatinine
	mm Hg	*μ*L/min/mc	*μ*g/mg	ng/mg	mIU/mg
DM					
Mean ± SD	173.36 ± 26.7	2.83 ± 1.16	66.5 ± 44.9	46.73 ± 31.59	0.87 ± 0.27
Median (range)	165.00 (145.00–227.00)	2.68 (1.52–4.63)	39.3 (23.4–133.3)	31.94 (25.04–109.59)	0.81 (0.57–1.38)
EN					
Mean ± SD	160.50 ± 26.80	2.9 ± 0.80	40.3 ± 17.5	37.05 ± 11.88	0.81 ± 0.24
Median (range)	164.25 (122.00–202.00)	3.07 (1.57–3.94)	34.3 (20.7–70.3)	32.09 (24.77–61.43)	0.71 (0.58–1.32)
LOS					
Mean ± SD	164.90 ± 18.64	3.29 ± 0.85	46.1 ± 24.0	30.77 ± 8.29	0.75 ± 0.35
Median (range)	166.50 (135.00–192.00)	3.02 (2.37–4.46)	44.4 (13.2–98.2)	28.72 (18.82–44.97)	0.64 (0.41–1.48)
EN + LOS					
Mean ± SD	160.15 ± 21.81	3.98 ± 0.97	49.2 ± 29.6	29.45 ± 18.28	0.66 ± 0.25
Median (range)	164.50 (110.50–180.00)	4.26 (2.11–5.31)	35.8 (24.9–111.1)	24.00 (15.17–81.20)	0.56 (0.29–1.11)
SPIR					
Mean ± SD	168.95 ± 32.76	3.07 ± 0.73	41.2 ± 15.2	36.12 ± 7.67	0.71 ± 0.22
Median (range)	159.50 (120.00–222.00)	3.04 (1.52–4.07)	37.4 (17.6–74.1)	32.50 (29.70–49.70)	0.66 (0.40–1.04)
DIH					
Mean ± SD	159.00 ± 15.67	2.9 ± 0.39	39.3 ± 19.8^*^	37.57 ± 12.84	0.73 ± 0.29
Median (range)	159.75 (139.00–190.50)	2.82 (2.46–3.53)	32.8 (16.9–72.1)	37.06 (20.67–58.45)	0.70 (0.21–1.21)

ANOVA test	NS	NS	*P* = 0.049	NS	NS

The results are presented as mean ± SD and median (range). Statistically significant differences between the groups have been shown, the levels of significance *P* ≤ 0.05 in the 95% confidence interval. ^*^ Statistical significance with the DM group.

**Table 3:** The values of body weight and kidney weight and ratio of kidney weight/body weight and FN concentrations and cathepsin B activity in homogenates of glomeruli and the content of fibronectin within the glomerulus identified by immunohistochemistry.

Groups	Glomerular homogenates						Immunohistochemistry
	Kidney weight/body weight %	Protein/DNA	FN/protein	FN/DNA	Cathepsin B/protein	Cathepsin B/DNA	FN score
		*μ*g/*μ*g	ng/*μ*g	ng/*μ*g	*μ*IU/*μ*g	*μ*IU/*μ*g	
DM							
Mean ± SD	0.60 ± 0.06^**^	23.2 ± 6.50	0.054 ± 0.020	0.95 ± 0.28	1.36 ± 0.37^#$^	30.60 ± 9.65^#^	2.33 ± 0.52^$^
Median (range)	0.6 (0.53–0.68)	23.94 (14.64–32.89)	0.05 (0.029–0.088)	0.89 (0.68–1.48)	1.31 (0.92–1.79)	28.37 (21.09–50.96)	2.00 (2.00-3.00)
EN							
Mean ± SD	0.61 ± 0.03^**^	18.1 ± 6.00	0.034 ± 0.018	0.50 ± 0.28	1.5 ± 0.78^#$^	25.99 ± 16.75^#$^	2.0 ± 0.82^$^
Median (range)	0.6 (0.58–0.66)	16.85 (9.10–27.30)	0.031 (0.012–0.069)	0.42 (0.16–1.02)	1.43 (0.58–2.46)	19.14 (12.53–59.90)	2.00 (1.00–3.00)
LOS							
Mean ± SD	0.64 ± 0.05^$^^**^	15.68 ± 5.71^*^	0.056 ± 0.022	0.75 ± 0.30	1.39 ± 0.72^#$^	21.71 ± 14.32^#$^	1.60 ± 1.07
Median (range)	0.64 (0.57–0.75)	15.27 (7.75–25.19)	0.055 (0.016–0.09)	0.79 (0.24–1.10)	1.21 (0.49–2.97)	17.98 (10.71–49.64)	2.00 (0.00–3.00)
EN + LOS							
Mean ± SD	0.65 ± 0.11^**^	17.65 ± 5.99	0.058 ± 0.016	0.81 ± 0.33	3.11 ± 1.36^*^	51.79 ± 20.37^*^	2.30 ± 0.67^$^
Median (range)	0.61 (0.57–0.92)	17.73 (10.82–30.62)	0.058 (0.036–0.086)	0.80 (0.50–1.54)	2.78 (1.04–5.32)	53.96 (21.49–80.01)	2.00 (1.00–3.00)
SPIR							
Mean ± SD	0.59 ± 0.06^**^	17.31 ± 4.20^*^	0.058 ± 0.022	0.72 ± 0.27	2.58 ± 1.19^*^	44.88 ± 23.13^**^	0.90 ± 0.99^∗#^
Median (range)	0.59 (0.47–0.67)	16.64 (11.58–24.49)	0.057 (0.021–0.091)	0.70 (0.33–1.13)	2.78 (1.04–4.09)	49.28 (16.59–83.64)	1.00 (0.00–3.00)
DIH							
Mean ± SD	0.54 ± 0.05^*^	18.57 ± 3.90	0.062 ± 0.022	0.81 ± 0.38	1.17 ± 0.64^#$^	22.70 ± 14.97^#$^	1.70 ± 0.95
Median (range)	0.56 (0.47–0.6)	19.45 (12.91–22.77)	0.054 (0.04–0.097)	0.62 (0.45–1.50)	0.96 (0.52–2.29)	16.02 (7.66–51.97)	1.50 (1.00–4.00)

Results are shown as mean ± SD and median (range). Statistically significant differences between the groups have been shown, the levels of significance P ≤ 0.05 in the 95% confidence interval. ^*^Statistical significance with the DM group; ^#^statistical significance with the EN + LOS group; ^$^statistical significance with the SPIR group; ^**^statistical significance with the DIH group.

Kidney weight/body weight P value: DM versus DIH P = 0.01; DIH versus EN P = 0.01; DIH versus LOS P = 0.0001; DIH versus EN + LOS P = 0.01; DIH versus SPIR P = 0.04; SPIR versus LOS P = 0.03.

Protein/DNA P value: DM versus LOS P = 0.01; DM versus SPIR P = 0.045.

Cathepsin B/protein P value: DM versus EN + LOS P = 0.001; DM versus SPIR P = 0.02; EN + LOS versus EN P = 0.002; EN + LOS versus LOS P = 0.001; EN + LOS versus DIH P = 0.0004; SPIR versus EN P = 0.03; SPIR versus LOS P = 0.02; SPIR versus DIH P = 0.009.

Cathepsin B/DNA P value: DM versus EN + LOS P = 0.04; EN + LOS versus EN P = 0.01; EN + LOS versus LOS P = 0.01; EN + LOS versus DIH P = 0.01; SPIR versus EN P = 0.05; SPIR versus LOS P = 0.02; SPIR versus DIH P = 0.03.

FN by immunohistochemistry P-value: DM versus SPIR P = 0.003; SPIR versus EN P = 0.02; SPIR versus EN + LOS P = 0.003.
